# Evaluation of the efficacy of DDT indoor residual spraying and long-lasting insecticidal nets against insecticide resistant populations of *Anopheles arabiensis* Patton (Diptera: Culicidae) from Ethiopia using experimental huts

**DOI:** 10.1186/1756-3305-7-131

**Published:** 2014-03-28

**Authors:** Abebe Asale, Yehenew Getachew, Weriessaw Hailesilassie, Niko Speybroeck, Luc Duchateau, Delenasaw Yewhalaw

**Affiliations:** 1 Department of Biology, College of Natural Sciences, Jimma University, Jimma, Ethiopia; 2 Department of Horticulture and Plant Science, College of Agriculture and Veterinary Medicine, Jimma University, Jimma, Ethiopia; 3 Department of Health Sciences, Addis Ababa Science and Technology University, Addis Ababa, Ethiopia; 4 Institute of Health and Society (IRSS), Université Catholique de Louvain, Brussels, Belgium; 5 Department of Comparative Physiology and Biometrics, Faculty of Veterinary Medicine, Ghent University, Ghent, Belgium

**Keywords:** Ethiopia, *An. arabiensis*, Insecticide resistance, Experimental huts, Long-lasting insecticide treated nets

## Abstract

**Background:**

Indoor Residual Spraying (IRS) and Long-Lasting Insecticidal nets (LLINs) are major malaria vector control tools in Ethiopia. However, recent reports from different parts of the country showed that populations of *Anopheles arabiensis*, the principal malaria vector, have developed resistance to most families of insecticides recommended for public health use which may compromise the efficacy of both of these key vector control interventions. Thus, this study evaluated the efficacy of DDT IRS and LLINs against resistant populations of *An. arabiensis* using experimental huts in Asendabo area, southwestern Ethiopia.

**Methods:**

The susceptibility status of populations of *An. arabiensis* was assessed using WHO test kits to DDT, deltamethrin, malathion, lambda-cyhalothrin, fenitrothion and bendiocarb. The efficacy of LLIN (PermaNet® 2.0), was evaluated using the WHO cone bioassay. Moreover, the effect of the observed resistance against malaria vector control interventions (DDT IRS and LLINs) were assessed using experimental huts.

**Results:**

The findings of this study revealed that populations of *An. arabiensis* were resistant to DDT, deltamethrin, lambda-cyhalothrin and malathion with mortality rates of 1.3%, 18.8%, 36.3% and 72.5%, respectively but susceptible to fenitrothion and bendiocarb with mortality rates of 98.81% and 97.5%, respectively. The bio-efficacy test of LLIN (PermaNet® 2.0) against *An. arabiensis* revealed that the mosquito population showed moderate knockdown (64%) and mortality (78%). Moreover, mosquito mortalities in DDT sprayed huts and in huts with LLINs were not significantly different (p > 0.05) from their respective controls.

**Conclusion:**

The evaluation of the efficacy of DDT IRS and LLINs using experimental huts showed that both vector control tools had only low to moderate efficacy against *An. arabiensis* populations from Ethiopia*.* Despite DDT being replaced by carbamates for IRS, the low efficacy of LLINs against the resistant population of *An. arabiensis* is still a problem. Thus, there is a need for alternative vector control tools and implementation of appropriate insecticide resistance management strategies as part of integrated vector management by the national malaria control program.

## Background

Malaria is endemic in 90 countries in tropical and subtropical zones [1]. It remains one of the greatest health threats in sub-Saharan Africa with high mortality and morbidity especially in children under the age of five years. In 2012, there were globally about 219 million cases and an estimated 660,000 deaths due to malaria with about 90% of these cases occurring in Africa [1,2].

In Ethiopia, malaria is seasonal in most parts of the country, with unstable transmission resulting in malaria epidemics. Malaria incidence decreased between 2004 and 2008, but in recent years malaria admissions increased, with the highest rate observed in 2011. In the aforementioned year only, 1,480,360 cases were observed of which 814,547 (55%) were due to *Plasmodium falciparum* and 665,813 (45%) due to *P. vivax*. The disease prevalence varies across regional states ranging from 0.5% to 2.5% [1,3]. *An. arabiensis,* a member of the *An. gambiae* complex, is the major vector in the country. Other anophelines which occur in Ethiopia are *An. funestus* group, *An. pharoensis* and *An. nili. An. funestus* and *An. pharoensis* are considered to be secondary vectors [4,5].

Long-lasting insecticidal nets (LLINs), indoor residual spraying (IRS) and environmental management are the most widely used tools for malaria vector control [6-8]. Despite reports demonstrating the efficacy of both ITNs and IRS for curbing malaria incidence [9] insecticide resistance in malaria vectors threatens the success of malaria vector control programs in sub-Saharan Africa [10]. If current trends continue, insecticide resistance may compromise control as it did in the last era of malaria eradication in the 1950’s and 60’s [11]. Given the limited number of available insecticides, i.e., only 12 insecticides belonging to 4 classes of insecticides (pyrethroids, organophosphates, carbamates and organochlorine) for IRS, and only one insecticide class (pyrethroids) for ITNs [12] the resistance related to these insecticides has become a limiting factor for malaria vector control.

Following DDT resistance reports undermining malaria vector control efforts [13], the controversy around the use of DDT shifted the attention to the use of pyrethroids, which are considered to be less toxic to humans and other non-target organisms [14]. Despite pyrethroids displaying better exito-repellent properties and faster killing effects than other insecticide classes, resistance to pyrethroids has emerged, spreading rapidly and constituting a serious threat to malaria control initiatives [15].

In Ethiopia LLINs & IRS are the two key vector control interventions. However, insecticide resistance, which became widespread in malaria vectors in western, southern, central and eastern Africa in recent years [16-19], is a major challenge in malaria vector control. *An. arabiensis,* has developed resistance against most insecticide families (organochlorines, organophosphates and pyrethroids) commonly used in public health [20-22]. The West African *kdr* (L1014F) mutation was also reported in populations of *An. arabiensis* from the different parts of the country with an allelic frequency of 95–100% [23-25]. Moreover, pre-exposure of *An. arabiensis* from southwestern Ethiopia to piperonylbutoxide (PBO) significantly increased the susceptibility of the population to both permethrin and deltamethrin, indicating the possible involvement of metabolic resistance in addition to the previously described *kdr* resistance [26]. DDT spraying was discontinued in 2009 and was replaced by deltamethrin. Furthermore, in 2012 Ethiopia switched from deltamethrin to bendiocarb for IRS in response to the observed resistance.

Despite the current high coverage of IRS and scaling up of LLINs, there is no documented information yet on the effect of insecticide resistance on the existing malaria vector control interventions in Ethiopia. Thus, the objective of this study was to evaluate the efficacy of DDT IRS and LLIN (PermaNet 2.0) on the control of DDT and pyrethroid resistant populations of *An. arabiensis* from Ethiopia in terms of deterrence, exit rate, blood feeding inhibition, mortality and personal protection using experimental huts.

## Methods

### Study area and period

This study was conducted from August to November 2011 around the Gilgel-Gibe hydropower dam area, southwestern Ethiopia. The Gilgel-Gibe hydroelectric power dam is one of the largest hydropower dams in Ethiopia. It produces about 184 MW and is located 260 km south west of Addis Ababa, in Oromia regional state, southwestern Ethiopia. It has become operational in 2004. The region is located between latitudes 7°42′50″N and 07°53′50″N and longitudes 37°11′22″E and 37°20′36″E, at an altitude ranging from 1,672-1,864 m above sea level. The region has a sub-humid, warm to hot climate, receives between 1,300 and 1,800 mm of rain annually and has a mean annual temperature of 19°C. The rainfall is divided in the long rainy season starting in June and extending up to September, and the short rainy season beginning in March and extending to April/May.

### Insecticide bioassays

Anopheline mosquito larvae were collected by dipping from a range of breeding sites (road paddies, brick pits, pools, marshes, streams, surface water harvest, ditches, dam reservoir shore, and pits dug for plastering traditional tukuls), around Osso Bille village, Asendabo, where the experimental huts were established. Mosquito larvae were reared to adults in the field Vector Biology Laboratory, Jimma University under standard conditions (temperature 25 ± 2°C, relative humidity 80 ± 4%). The larvae were fed with dog biscuits and brewery yeast [27]. Two to three days old, non-blood-fed female mosquitoes were exposed to insecticide impregnated papers using the insecticides DDT (4%), deltamethrin (0.05%), malathion (5%), lambda-cyhalothrin (0.05%), fenitrothion (1.0%) and bendiocarb (0.1%), following the WHO standard assay [28,29]. The insecticide impregnated and control papers were obtained from the WHO collaboration Centre, Vector Control Research Unit, School of Biological Sciences, Penang, Malaysia. Eighty mosquitoes in 4 replications (20 mosquitoes per replicate) were exposed in test kit tubes for one hour for each of the five insecticides except fenithrothion and knockdown was recorded for DDT, deltamethrin and lambda-cyhalothrin at 10, 15, 20, 30, 40, 50, and 60 minutes. For fenitrothion, 84 mosquitoes in 4 replications (20–22 mosquitoes per replicate) were exposed in test tubes for two hours. An equal number of mosquitoes (two replicates each with 20 mosquitoes) were exposed to the corresponding control papers impregnated with resila oil (Organochlorine control), olive oil (organophosphate/carbamate control), and silicone oil (pyrethroid control). Following exposure, mosquitoes were transferred into holding tubes and provided with 10% sucrose solution with cotton pads. Mortality was recorded 24 hours post exposure. Moreover, the quality of the insecticide impregnated papers was assessed using the susceptible colony of *An. arabiensis* strain from The WHO Malaria Training Center, Nazareth, Ethiopia and the susceptible strain was fully susceptible to all tested insecticides with mortality ranging from 98.5-100%.

### LLIN sample preparation and WHO cone assays

Three rectangular nets of PermaNet® 2.0 and three untreated nets to be used as a negative control were purchased from the local market in Ethiopia. The production date and batch number of all nets were recorded. Three sub-samples per net (one from the roof and two from each long side of the net) were taken from each net and prepared for standard LLINs cone tests by cutting 30 cm × 30 cm pieces. Each sub-sample was rolled up in aluminum foil, labeled (by net type, net number and sample area) and kept individually in a refrigerator prior to the assay. For each individual sub-sample, four cone tests were conducted sequentially following the standard WHO procedure [30]. Five non blood-fed, two to three days old, female mosquitoes were introduced into each cone and exposed to each bed net sample for 3 minutes before being transferred to paper cups and held with access to 10% sugar solution. Knockdown (KD) was recorded at 1, 3, 5, 10, 15, 30, 45 and 60 minutes and mortality (MT) was recorded 24 hours post-exposure. A total of 180 mosquitoes were tested (20 mosquitoes × 3 subsamples × 3 nets). Replicates of cone assays with sub-samples taken from untreated nets were also conducted concurrently as a negative control. Mortality was corrected using Abbott’s formula when mortality in the control exceeded 5% [31]. Bioassays were carried out at a temperature of 27 ± 2°C and relative humidity of 80 ± 4%.

### Establishment of experimental huts

**Figure 1:**
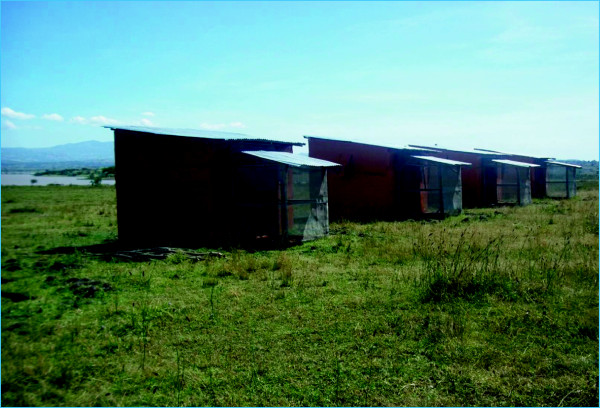
Experimental huts used for the study.

Four experimental huts, each with one room and a large screened veranda trap were established approximately 500 m West of the Gilgel-Gibe reservoir shore, southwestern Ethiopia, and used for the evaluation of the efficacy of IRS and LLIN [Figure 1]. The experimental huts were constructed following the WHO guidelines [30]. The dimensions of the hut were 2.5 m wide, 2.5 m long and 3 m high while that of the verandah trap was 2 m long, 1.5 m wide and 1.5 m high being projected from the back wall of each hut. The walls of the huts were constructed from plywood and wooden frame for easy manipulation and transportation. The huts were covered with red brown colored polyethylene plastic on the outside in order to simulate the wall color of local tukuls. The roof was made of corrugated iron sheet. The slits were constructed from pieces of plywood, fixed at an angle of 45° to create a funnel of 1 cm between slits. The window slits were designed in such a way that the mosquitoes could not escape once they entered the hut. The window slits were made in such a way to allow those mosquitoes fly upward to enter into the huts through the open space and those which fly downward to exit; consequently, the design of the slits precluded influx of mosquitoes into and out of the experimental huts. Each hut had a veranda trap made of iron meshes (22 mm) for trapping exophilic mosquitoes. Mosquitoes inside the hut could only exit via the veranda, which was shut down by lowering a curtain separating the sleeping room from the veranda. Each hut had a ceiling made of white sheets. A gutter was dug around each hut and filled with water to exclude ants and other scavenger arthropods which otherwise could carry off dead mosquitoes from the huts during the night. Each night white sheets were spread on the floor of the experimental hut to collect knocked down and/or dead mosquitoes.

### Treatment arms and sleepers rotation

The treatments for this trial were DDT for IRS and PermaNet 2.0 for LLINs. DDT was obtained from Adami Tulu Pesticide Processing S.C. Addis Ababa, Ethiopia and a WHOPES approved LLIN (PermaNet® 2.0) made of multifilament polyester fibers, factory-coated with a wash resistant formulation of deltamethrin at a target dose of 55 mg/m^2^ was obtained from a local market. A dose of 2 g/m^2^ DDT wettable powder (WP) was sprayed onto interior walls of one of the four huts, randomly chosen, using a Hudson compression sprayer equipped with a flat fan nozzle [29]. The untreated bed net is made of white 100-denier polyester multifilament net (Siamdutch Mosquito Netting Co., Ltd, Bangkok, Thailand). Six holes of 4 cm × 4 cm were made in each mosquito net, two in each long side and one at each shorter side to simulate the conditions of a torn net and to ensure that the insecticide, rather than the net, effectively prevents mosquito bites. Huts assigned for IRS treatment were fixed throughout the study according to the WHO guideline, as the IRS treatment could not be rotated due to residual effects of DDT [30]. The LLIN, untreated net and unsprayed hut treatments, however, were rotated weekly between huts, in a 3x3 Latin square design (LSD), with week and hut being the rows and the columns of the Latin square.

A baseline study was conducted in July, 2011 to evaluate the attractiveness of the experimental huts. The trial lasted for four weeks from July 20th, 2011 to August 24th, 2011. Eight teams of two people served as volunteer sleepers and each team was rotated between treatments on successive nights within a week to avoid possible bias that could arise due to individual attractiveness to mosquitoes. The teams slept in the huts from 19:00 h to 06:00 h each night. The sleepers were allowed to spend 2 days between rotations to clean and ventilate the huts before starting the next rotation to avoid possible contamination. The trial was conducted for eight weeks or 40 nights. Informed written consent was obtained from each sleeper.

### Mosquito collection, identification and determination of IRS and LLIN efficacy

Anopheline mosquitoes were collected each morning from 06:00 h to 7:00 h from inside bed nets, floors, walls, ceilings and veranda traps of each experimental hut using mouth aspirators and torches. Then the collected mosquitoes were recorded as dead or alive. Live mosquitoes were held in paper cups and supplied with 10% sucrose solution. The collected mosquitoes were transported to Asendabo Vector Biology Laboratory, Jimma University, where mosquitoes were sorted by genus, sex and morphologically identified using taxonomic keys [32]. Mosquitoes were also scored for their physiological state as unfed, fed, half gravid and gravid. Delayed mortality was recorded after 24 h.

To evaluate the efficacy of ITNs and IRS against the resistant population of *An. arabiensis*, different entomological parameters (deterrence, exit, blood feeding inhibition and mortality rates) were derived from basic measurements following an established formula [30].

The basic measurements considered were: number of collected female mosquitoes, blood-fed female mosquitoes and dead female mosquitoes, denoted respectively by N, B, and D. These basic measurements were indexed to denote the collection place (first sub-index) and the treatment (second sub-index). For location, ‘h’ refers to collection from inside the hut, whereas ‘e’ refers to the verandah trap, and finally ‘t’ is the sum of the two (‘h’+’e’).

For treatment, ‘c’ refers to unsprayed hut, ‘i’ to sprayed hut (IRS), ‘u’ to untreated bed net and ‘b’ to treated bed net (LLIN).

In comparing IRS with its control, the deterrence rate for IRS is given by

$$\mathrm{Deterrence}\;\mathrm{rate}\;\mathrm{IRS}=100\times \frac{\left(\mathrm{Nt},c-\mathrm{Nt},I\right)}{\mathrm{Nt},c}$$

N_t,c_ = the sum total no of mosquitoes collected from a hut and exit trap of unsprayed hut

N_t,I_ = the sum total no of mosquitoes collected from a hut and exit trap of sprayed hut

whereas the deterrence rate for treated LLIN compared to its control is given by

$$\mathrm{Deterrence}\;\mathrm{rate}\;\mathrm{LLIN}=100\times \frac{\left(\mathrm{Nt},c-\mathrm{Nt},I\right)}{\mathrm{Nt},c}$$

N_t,c_ = the sum total no of mosquitoes collected from a hut and exit trap with untreated net

N_t,I_ = the sum total no of mosquitoes collected from a hut and exit trap with LLIN

For a particular hut with treatment j, the entomological parameters are defined as

$$\mathrm{Exit}\;\mathrm{rate}=\frac{N_{e,j}}{N_{t,j}}\times 100$$

$$\mathrm{Blood}\;\mathrm{feeding}\;\mathrm{inhibition}\;\mathrm{rate}=\frac{B_{t,j}}{N_{t,j}}\times 100$$

$$\mathrm{Mortality}\;\mathrm{rate}=\frac{D_{t,j}}{N_{t,j}}\times 100$$

$$\mathrm{personal}\;\mathrm{protection}\;\left(\%\right)=100\times \frac{\left(\mathrm{Bc}‒\mathrm{Bt}\right)}{\mathrm{Bc}}$$

B_c_ = total no of blood-fed mosquitoes in the hut with untreated net

B_t_ = total no of blood-fed mosquitoes in hut with LLIN

$$\mathrm{Killing}\;\mathrm{effect}\;\left(\%\right)=100\times \frac{\left(\mathrm{Dt}‒\mathrm{Dc}\right)}{\mathrm{Ec}}$$

D_t_ = total no of mosquitoes dead in a hut with LLIN

D_c_ = total no of mosquitoes dead in a hut with untreated net

E_c_ = total no of mosquitoes entering a hut with untreated net

### Ethical consideration

The study protocol was reviewed and approved by the research and ethics committee of Jimma University, Ethiopia. The volunteers were provided with mefloquine as chemoprophylaxis as per the national malaria treatment guideline of Ethiopia and each volunteer was monitored every other day for fever. However, the volunteers/sleepers were not vaccinated against yellow fever as there was no previous reports of yellow fever infection in the study area.

### Data analysis

The LLIN and untreated bed net on the one hand, and sprayed and unsprayed hut on the other hand, were compared with one another with respect to blood feeding inhibition, exit and mortality rates. A linear fixed effects model was used including treatment and week as fixed effects. F-tests were performed at a global significance level of 5% but testing each of the two comparisons at the Bonferroni adjusted comparisons wise significance level of 2.5%. All analyses were done using SAS software package version 9.3 (SAS Institute Inc., Cary, NC, USA).

## Results

### Insecticide and cone bioassays

**Figure 2:**
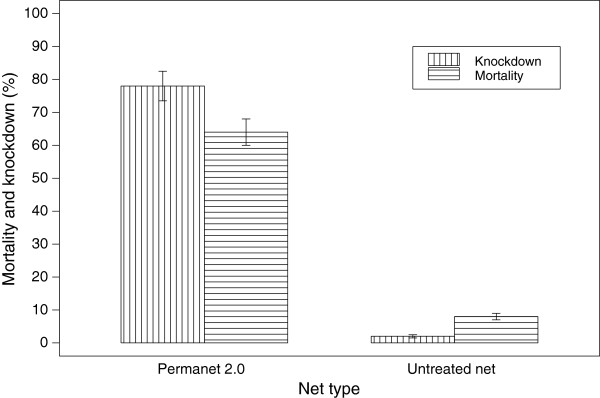
Mean percent mortality and knock down (SE) of WHO cone bioassay test for PermaNet® 2.0 and Untreated Net, July - August, 2011, Jimma, Southwestern Ethiopia.

The susceptibility status of populations of *An. arabiensis* to five insecticides commonly used in malaria vector control in Ethiopia is shown in Table 1. Populations of *An. arabiensis* showed reduced mortality to DDT, deltamethrin, lambda-cyhalothrin and malathion; however, the mosquito population was fully susceptible to fenitrothion and bendiocarb. It was impossible to calculate KT_50_ and KT_95_ for pyrethroids (deltamethrin & lambda-cyhalothrin) following the insecticide bioassay as only few mosquitoes were knocked down (only 8 mosquitoes after 50 minutes for lambda-cyahalothrin and 26 mosquitoes after 40 minutes for deltamethrin). Exposure of mosquitoes to net sections of PermaNet® 2.0 in cone bioassay tests led to an observed average mortality of 64% and knock down of 78%, which is well below the required levels of 80% and 95%, respectively (Figure 2). The target concentration for the LLIN (PermaNet 2.0) fell within the manufacturer specifications as determined by HPLC (range 41.25-68.75 mg/m^2^) [26].

**Table 1:** **Mean mortality rate of *An. arabiensis *** **to six insecticides, southwestern Ethiopia**

**Type of insecticide**	***An. arabiensis *** **tested**			***An. arabiensis *** **control**		
	**No. tested**	**No. dead**	**% Mortality**	**No. tested**	**No. dead**	**% Mortality**
DDT (4%)	80	1	1.25	40	0	0
Deltamethrin (0.05%)	80	15	18.75	40	0	0
Malathion (5%)	80	58	72.50	40	2	5
Lambdacylothrin (0.05%)	80	29	36.25	40	0	0
Fenitrothion (1.0%)	84	83	98.81	40	0	0
Bendiocarb (0.1%)	80	78	97.50	40	0	0

### Mosquito deterrence rate, personal protection and insecticidal effect

Overall, 2391 and 1023 anopheline and culicine mosquitoes were collected, respectively during the trial. Of the 2391 anopheline mosquitoes, 2209 (92.4%) belonged to *An. gambiae* s.l., presumably *An. arabiensis* Yewhalaw *et al*. [19,24], 160 (6.7%) to *An. coustani* and 22 (0.9%) to *An. pharoensis*. Of the total 2209 *An. arabiensis* collected, 479 (22%) were from the DDT sprayed hut, 793 (36%) were from the unsprayed hut, 426 (19%) from huts with LLIN and the remaining 511 (23%) from the hut with an untreated net. The deterrence rate of the DDT sprayed hut and a hut with LLIN was 39.6% and 16.6%, respectively. Moreover, personal protection in a hut with LLIN was over 21% against *An. arabiensis* as compared to a hut with untreated nets while the insecticidal effect in a hut with LLIN was 19.6%.

### Mosquito mortality, blood feeding and exit rates

Mosquito blood feeding rates, exit rates and mortality rates of the 4 treatments are presented in Table 2. There was no significant difference (p > 0.05) in mosquito blood feeding rates between the sprayed (76.1%) and unsprayed hut (80.3%) and between a hut with a treated net (55.1%) and the hut with an untreated net (58.9%). Moreover, the mean exit rate was similar (P > 0.05) for the sprayed hut (48.6%) and unsprayed hut (42.3%) and between a hut with treated net (49.4%) and a hut with untreated net (41.4%). There was no significant (P > 0.05) difference in mosquito mortality between the sprayed and unsprayed hut, or between a hut with LLIN and a hut with untreated net.

**Table 2:** **Mean blood feeding, exit rate and mortality rate of *An. arabiensis***

**Treatment**	**Blood feeding rate**	**Exit rate**	**Mortality rate**
	**n (Mean ± SE)**	**n (Mean ± SE)**	**n (Mean ± SE)**
Sprayed hut	364 (76.1 ± 5.1)	233 (48.6 ± 3.9)	247 (51.5 ± 5.6)
Unsprayed hut	641 (80.8 ± 6.6)	335 (42.3 ± 4.8)	324 (40.8 ± 5.5)
Hut with LLIN	235 (55.14 ± 3.9)	210 (49.4 ± 4.8)	247 (58.0 ± 7.0)
Hut with untreated net	301 (58.90 ± 5.7)	211 (41.4 ± 5.2)	294 (57.50 ± 6.7)

## Discussion

Insecticide resistance is a major impediment in malaria vector control. In this study we initially assessed the susceptibility status of field population of *An. arabiensis* using WHO susceptibility test kits and bio-efficacy of LLINS. We further assessed the impact of resistance on the existing vector control interventions (IRS & LLINs) using experimental hut trials following the WHOPES guidelines [30]. The results of the WHO insecticide susceptibility test showed that populations of *An. arabiensis* developed resistance to DDT, deltamethrin, malathion, and lambda-cyhalothrin but were still susceptible to fenitrothion and bendiocarb. Previous reports from Ethiopia also showed that *An. arabiensis* populations have developed resistance against three classes of insecticides. Yewhalaw *et al.*[19,24,26] reported that populations of *An. arabiensis* from southwestern Ethiopia had developed resistance to DDT, Permethrin, deltamethrin, and malathion but were fully susceptible to propoxur. A similar study by Balkew *et al.*[20] in villages of central, northern and south western Ethiopia showed that populations of *An.arabiensis* developed resistance to DDT, deltamethrin, lambda-cyhalothrin, malathion and Bendiocarb. Recently, Fetene *et al*. [25] reported that populations of *An. arabiensis* from southern and northern parts of the country were resistant to DDT and malathion. Another recent study conducted by Massebo *et al*. [21] around southern Ethiopia revealed that populations of *An. arabiensis* were resistant to lambda-cyhalothrin, cyfluthrin and alpha-cypermethrin, deltamethrin, and DDT. A similar study conducted by Abate and Hadis [22] in northern, northwestern, central and southern Ethiopia confirmed the development of high level pyrethroid and DDT resistance in populations of *An gambiae* s.l. Likewise a widespread pyrethroid resistance by *An. arabiensis* was reported from western Kenya [33]. In the same way a study carried out in two villages of Côte d’Ivoire, confirmed that resistance had developed at various degrees in both regions [34]. Similarly, insecticide susceptibility test reports from Burkina Faso, Chad and Sudan showed that all mosquito populations of *An. gambiae* s.l. from Burkina Faso and Chad and populations of *An. arabiensis* from Sudan were resistant to permethrin, deltamethrin, and DDT, whereas the same population remained largely susceptible to fenitrothion and bendiocarb [35].

The mortality and knockdown results from the WHO cone bioassay test revealed that unwashed PermaNet® 2.0 had a reduced efficacy, although it caused much higher mortality and knockdown rates compared to the untreated net. Previous studies from the same region showed that *An. arabiensis* populations have developed pyrethroid resistance [26]. The involvement of metabolic resistance in populations of *An. arabiensis* had been reported using synergists [26]. Norris and Norris [36] reported that *An. arabiensis* populations in Zambia showed resistance to DDT and 12% of the mosquitoes tested survived after exposure to ITNs. In agreement with this finding, populations of *An. arabiensis* from Tanzania [37] showed resistance to PermaNet® 2.0 with mortality reduced from 92.8% in the first month to 83.3% after six months. Similar results were reported from a study carried out in Côte d’Ivoire [38] with wild resistant *An. gambiae* mosquitoes showing a mean knockdown and mean mortality rates below 95% and 80%, respectively for all treatment arms, with the exception of unwashed PermaNet® 3.0 which caused knock down and mortality rates of 95.8% and 97.0%, respectively.

There was a 39.6% reduction in deterrence rate of *An. arabiensis* in DDT sprayed huts as compared to unsprayed huts and a reduction of 16.6% of mosquito deterrence rate in huts with LLIN as compared to huts with untreated nets. Similarly a study conducted in Tanzania using experimental hut trials revealed that PermaNet® 2.0 resulted in a 21% reduction in deterrence rate of *An. arabiensis*[39]. Another study from Burkina Faso using experimental huts documented that the entry rate of *An. gambiae* s.s. into huts with LLIN and insecticide treated plastic sheeting (ITPS) was reduced as compared to untreated huts [40]. Moreover, a study conducted in Vietnam using experimental huts revealed a 30.7% reduction in populations of *An. epiroticus* density entering into huts treated with PermaNet® 2.0 [41].

The mosquito feeding and exit rates were very similar in the sprayed and unsprayed huts, and also in the hut with LLIN and a hut with an untreated net. This is consistent with the findings of Ngufor *et al.*[42] from Benin who showed that induced exophily rates in *An. gambiae* s.s. between the huts with LLIN (PermaNet® 2.0) and CTN compared to their untreated controls were similar. Corbel *et al.*[43] also noted the absence of significant reduction in entry rate between LLIN and untreated nets in their experimental hut study in the village of Malanville, Benin. In our study, mosquito mortality rates between the sprayed hut and its control, and between a hut with LLIN (PermaNet® 2.0) and a hut with untreated net were similar. In contrast to this, a study conducted in Côte d’Ivoire showed that both unwashed PermaNet® 2.0 and PermaNet® 3.0 caused significantly higher mosquito mortality as compared to their respective control [38]. A study from Vietnam also showed significantly higher mosquito mortality among the treatment arms (huts treated with PermaNet® 2.0, PermaNet® 3.0 and CTN) as compared to their control [41].

## Conclusion

In conclusion, populations of *An. arabiensis* around the Gilgel- Gibe dam area, south western Ethiopia have developed resistance to organochlorines, organophosphates, and pyrethroids. The evaluation of IRS using DDT and LLINs (PermaNet® 2.0) based on a trial with experimental huts further suggests that neither DDT nor LLIN can stand alone as a vector control tool in the presence of the resistant mosquito population in the study region. Therefore, alternative new vector control tools should be put in place and an insecticide resistance management strategy plan should be developed and implemented. One possible option could be combining LLIN with IRS using a new insecticide of choice (e.g., bendiocarb), which could reduce vector-human contact in the study area. Furthermore, large scale field trial studies should be carried out in order to confirm whether the current vector control interventions, IRS and LLINs, are still effective in different regions of Ethiopia in the presence of resistant populations of *An. arabiensis*.

## Competing interests

The authors declare that they have no competing interests.

## Authors’ contributions

AA and YD conceived and designed the study, were involved in supervision of data collection and drafted the manuscript. WH was involved in field data collection. YG and NS analyzed the data. LD was involved in data analysis and critically reviewed the manuscript. All authors read and approved the final manuscript.
